# Supplementary material for: Optimizing Black Soldier Fly frass fertilizer rates for maize and soybean production in Madagascar
Source: PLoS One. 2026 May 4;21(5):e0343378. doi: 10.1371/journal.pone.0343378 (PMC13138682; doi:10.1371/journal.pone.0343378)
Supplement: Attachment — Submitted filename: Major & Minor Comments.docx [file pone.0343378.s004.docx]

**Major Comment**

This manuscript presents a well-designed field study evaluating the agronomic and economic performance of BSF frass as an organic fertilizer for maize and soybean in Madagascar. The study addresses a critical constraint to agricultural intensification in low-income systems, namely fertilizer affordability, and integrates asymptotic yield modeling with NAE and MVCR analyses. The results demonstrate consistent yield increases without evidence of yield penalties at high application rates and provide an agronomic–economic framework that could inform fertilizr recommendations under resource-constrained conditions. Beyond its immediate findings, the study has practical relevance for circular bioeconomy strategies, offering evidnce that locally produced organic fertilizers such as BSF frass may help reduce dependency on imported mineral fertilizers, enhance nutrient recycling, and contribute to narrowing yield gaps in smallholder systems.

However, the experimental design indicates that dolomite was systematically applied to half of the plots within each frass rate, effectively creating a factorial structure, yet dolomite was not included as a fixed factor in the main statistical model, nor were frass × dolomite interactions formally explained. Given the well-established effects of liming on soil pH, nutrient availability, and nitrogen dynamics, excluding this factor may influence interpretation of the dose–response relationships. In addition, flooding events were reported but not clearly incorporated or analytically justified in the primry model. For consistency between experimental design and statistical inference, these factors should either be explicitly modeled (including dolomite and its interaction with frass rate in a mixed-effects framework) or clearly reported as tested and found non-significant, with appropriate model specifications and statistical evidence. That said, I remain neutral at this stage, as relevant data are presented in the Supplementary Material. I would therefore be interested to see whether the second reviewer shares similar concerns before recommending a more definitive course of action.

In addition, I consider it important to highlight that several sections, particularly within the Methods, contain limited citation of supporting literature. Methodological decisions should ideally be grounded in established experimental design principles or prior validated approaches, yet the manuscript provides relatively few references to justify key analytical and experimental choices. This limited citation base may compromise methodological traceability and future reproducibility. I therefore strongly encourage the authors to substantially strengthen the reference support, especially in the Methods section, to enhance transparency, scientific rigor, and reproducibility.

Based on the considerations outlined above, I regret that at this stage I must recommend **major revision**. This decision reflects concerns regarding analytical consistency and methodological transparency rather than the overall scientific merit of the work. I strongly encourage the authors to address the issues raised, as the findings are important and have significant potential impact; with appropriate revisions, this study could make a valuable contribution to the literature and to broader discussions on sustainable fertilizr strategies in agricultural systems.

I have prepared a table to provide a more detailed overview of the specific points that require improvement based on my feedback, which is presented below as Table 1.

**Tabel 1. Minor Comment**

| No. | Line(s) | Comments |
| --- | --- | --- |
| 1 | 26-36 | Please add information on frass productivity derived from similar waste streams or from the local region, to illustrate product abundance and its potential as a fertilizer alternative. |
| 2 | 107-109 | Could you clarify whether the selection of these frass application rates is based on established references or prior studies? Please provide the supporting sources. |
| 3 | 117-127 | Please provide references to support the technical procedures used for maize and soybean planting. |
| 4 | 154, 157, 167, 194, 207 | Please provide references to support the technical procedures used for this study. |
| 5 | 224-225, 235-236 | Add definitions/explanations for the equation parameters, especially 'ton', 'ton h^-1^', etc. These should be clearly described. |
| 6 | 224 | Add a citation to “(Equation 1)” in the text after “Nitrogen agronomic efficiency (NAE) was calculated as”. |
| 7 | 223 | The formulation of nitrogen agronomic efficiency (NAE) requires clarification. Please provide the literature reference supporting the equation used, present the formula explicitly in its complete mathematical form (including the denominator), and clearly state all units for each term to avoid ambiguity. In addition, the definition of “NAE-adjusted yield” should be written as an explicit equation, with its corresponding units, and supported by an appropriate methodological or theoretical justification. Clear and transparent presentation of these formulas is essential for reproducibility and reader comprehension. |
| 8 | 234 | Add a citation to “(Equation 1)” in the text after “Economic performance was evaluated using the marginal value-cost ratio (MVCR), calculated as”. |
| 9 | 234-239 | Please provide an appropriate reference to support this formula. |
| 10 | 295, 300 | The reported application rate of 0.04 t ha⁻¹ associated with the highest NAE appears inconsistent with the described treatment range (0.4 t ha⁻¹ to 30 t ha⁻¹) and seems unusually low, therefore please clarify whether this value is correct, model-derived, or a typographical error. |
| 11 | 325-326 | Please provide a verifiable source for the local crop prices described as “approximately double the FAO values,” as these data are currently cited as unpublished; given that profitability thresholds scale directly with crop price, transparent documentation of these local price assumptions is essential for evaluating the robustness of the economic conclusions, and these data should be included either directly in the manuscript or as supplementary material. |
| 12 | 397-399 | Please provide the supporting literature for this statement, as no reference is currently cited to substantiate the statement. |
| 13 | 427-428 | The statement “substantially lower than those for cattle manure, mineral NPK fertilizer in Madagascar, and mineral fertilizers reported elsewhere in sub-Saharan Africa” lacks an explicit citation for cattle manure prices specifically in Madagascar. Reference [6] appears to cover mineral fertilizer cost comparisons but does not substantiate the claim regarding cattle manure. Please provide an appropriate literature or data source supporting the cattle manure price comparison, and include this reference directly in the manuscript or, if necessary, in the supplementary material. |
| 14 | Fig. 4 | In Figure 4, the contour labels (1, 2, 3, 10, 50) overlap with key areas of the plot and partially obscure the underlying data, making interpretation difficult; please adjust the placement of these labels to improve clarity and readability. |
| 15 | Fig. 4C | Figure 4C is not mentioned in the manuscript text. Please ensure it is appropriately referred to and its role in the study is clearly described |
| 16 | References | Please add more references from the recent five years to show the novelty/new progress brought by this study. There are still some from 2002, 2005, 2008, 2011, 2012, 2019. |

Sincerely,

Mulki Salendra Kusumah
