# Supplementary material for: Optimizing Black Soldier Fly frass fertilizer rates for maize and soybean production in Madagascar
Source: PLoS One. 2026 May 4;21(5):e0343378. doi: 10.1371/journal.pone.0343378 (PMC13138682; doi:10.1371/journal.pone.0343378)
Supplement: Attachment — Submitted filename: Response_to_Reviewers.docx [file pone.0343378.s007.docx]

Response to Reviewers

**Manuscript title:** Optimizing Black Soldier Fly frass fertilizer rates for maize and soybean production in Madagascar

**Dear Editor,**

Thank you for the thoughtful comments and for the opportunity to revise our manuscript. We have substantially revised the manuscript in response to the reviewers’ suggestions.

The most important change concerns the analytical treatment of dolomite application and flooding. After re-evaluating the dataset, we concluded that plots receiving dolomite were confounded by unintended differences in implementation, including a difference in the interval between fertilization and sowing. Because we could not confidently attribute any observed differences specifically to dolomite, we removed all dolomite-treated plots from the revised analyses. We also removed plots affected by the anomalous flooding event. Although this reduced the dataset, the main conclusions remained unchanged. We believe this substantially simplifies the manuscript and strengthens the inference regarding the effect of frass application rate.

We also agree with Reviewer #2 that the “NAE-adjusted optimum” was conceptually awkward. We therefore removed that analysis and all associated interpretation, and now focus on two clearer dimensions of inference: biophysical response and economic profitability.

In addition, we extensively revised the Discussion, strengthened the Methods, and added 27 references, including 11 published within the last five years. We also added a paragraph in the Introduction on frass productivity from organic waste streams, clarified the rationale for the fertilizer dose design, revised the economic interpretation to emphasize that the analysis represents a theoretical upper bound on economically viable frass prices, and improved methodological transparency throughout.

We hope that these revisions have improved the manuscript and made it suitable for publication in PLOS ONE.

Sincerely,

Tanjona Ramiadantsoa

# Reviewer #1

*Thank you for the submission. Even the paper is interesting, I have some comments before accepted this paper. Specific comments: Extend introduction with more refs. Give more information regarding data analyses. Discussion is superficial. Please give more data.*

**Response:** We thank the reviewer for these helpful comments. In response, we expanded the Introduction with additional references, clarified the Data Analyses section, and substantially revised the Discussion to provide more interpretation and context for the results. These revisions were also informed by the more specific suggestions of Reviewers #2 and #3.

# Reviewer #2

*This is a timely and relevant study that addresses a critical gap in the practical application of insect-based fertilizers. The large-scale field trial and the focus on both agronomic and economic optima are significant strengths. However, several points require clarification, correction, or further development to enhance the manuscript's rigor and impact before publication.*

**Response:** We thank the reviewer for the positive assessment of the study and for the constructive suggestions. We address each point below.

## 1. Dolomite application and flooding were not adequately accounted for in the original analyses.

*You have introduced two significant uncontrolled variables: flooding and dolomite application. By not including dolomite as a fixed factor in your statistical models and by not stratifying or blocking for the flooding effect, you cannot separate the true effect of the frass rate from the effects of liming and waterlogging stress. The primary analysis must account for these factors.*

**Response:** We agree that these factors required clearer treatment. After re-evaluating the dataset, we concluded that the dolomite treatment could not be interpreted cleanly because it was confounded by unintended implementation differences, most notably a three-day difference in the interval between fertilizer application and sowing. We therefore removed all dolomite-treated plots from the revised analyses rather than presenting an analysis that might be misleading. We also removed plots affected by the anomalous flooding event. In the revised dataset, 44 maize plots and 32 soybean plots were retained. We re-ran all analyses, and the main conclusions remained unchanged, except that soybean individual yield no longer clearly approached an asymptote. Overall, germination and yield responses were slightly stronger in the revised analyses. We believe this decision simplifies the manuscript and strengthens the inference regarding the effect of frass application rate.

## 2. NAE-adjusted yield should be removed.

*NAE is a descriptive metric for a specific rate. Multiplying it by the predicted yield to find a maximum is mathematically unusual and biologically ambiguous. The NAE-adjusted optimum is conceptually less clear than the MVCR analysis.*

**Response:** We agree and have removed the NAE-adjusted yield analysis and all associated interpretation from the revised manuscript. We now use NAE only as a descriptive efficiency metric and focus the main narrative on two clearer and complementary concepts: biophysical response and economic profitability.

## 3. The economic analysis should be reframed as a theoretical upper bound rather than a practical farm-level recommendation.

*The current analysis shows the maximum allowable frass price at the farm gate. It does not demonstrate that using these rates is profitable in reality because labor, transport, and other costs are not included.*

**Response:** We agree and revised the Methods, Results, and Discussion to make clear that the analysis provides a theoretical upper bound on economically viable frass prices rather than a complete farm-level profitability assessment. In the Methods, we now state: “A comprehensive assessment of farm-level profitability should ideally incorporate labor costs, transport expenditures, and logistical overheads, all of which are highly context-specific (e.g., Mamadou et al., 2020). Rather than offering prescriptive management recommendations, this study explores the theoretical economic boundaries of frass application, with a specific focus on fertilizer-to-crop price ratios.” We also added two paragraphs in the Discussion clarifying that FAO prices are only a reference point, that MVCR rescales proportionally with crop price, and that labor, transport, land tenure, and production structure all influence realized profitability.

## 4. Expand on the physiological trade-off observed in soybean and the role of initial soil phosphorus.

*Expand on the physiological trade-off observed in soybean (growth vs. yield) and the importance of the soil's initial P status for interpreting the results.*

**Response:** We expanded the Discussion with two new paragraphs addressing these points. First, we discuss the divergence between individual-level and plot-level soybean yield responses and interpret this pattern as a density-related trade-off in which vegetative growth becomes decoupled from reproductive output at high fertilizer rates. Second, we discuss the implications of the relatively high baseline soil phosphorus at the study site and clarify that the observed responses likely reflect primarily nitrogen and possibly other nutrients supplied by the frass. We also discuss how responses might differ in soils with lower baseline phosphorus.

## 5. Soil context and phosphorus availability.

*The positive yield response is likely driven largely by nitrogen rather than phosphorus because the soil already had relatively high available phosphorus. This should be highlighted.*

**Response:** We agree and now address this explicitly in the Discussion as part of the expanded paragraph on soil phosphorus context and generalizability.

## 6. Elaborate on the soybean growth–yield relationship shown in Figure 2D.

*The declining slope in soybean suggests that high early vegetative cover does not necessarily translate into high grain yield at high frass rates, possibly due to a trade-off favoring vegetative growth over pod filling.*

**Response:** We agree and expanded the Discussion accordingly. We now interpret the weakening growth–yield relationship at high soybean fertilizer rates as evidence that spatial competition and canopy expansion decouple vegetative growth from reproductive output under dense planting conditions.

# Reviewer #3

*This manuscript presents a well-designed field study evaluating the agronomic and economic performance of BSF frass as an organic fertilizer for maize and soybean in Madagascar. The study addresses a critical constraint to agricultural intensification in low-income systems, namely fertilizer affordability, and integrates asymptotic yield modeling with NAE and MVCR analyses. The results demonstrate consistent yield increases without evidence of yield penalties at high application rates and provide an agronomic–economic framework that could inform fertilizer recommendations under resource-constrained conditions.*

**Response:** We thank the reviewer for this positive assessment and for the detailed and constructive comments. We address the major and minor points below.

## Major comment. Dolomite and flooding should be handled more explicitly.

*The experimental design indicates that dolomite was systematically applied to half of the plots, yet dolomite was not included as a fixed factor in the main statistical model. Flooding events were also not clearly incorporated or analytically justified in the primary model.*

**Response:** We thank the reviewer for this important point. We shared this concern, especially after the same issue was raised by Reviewer #2. After re-evaluating the dataset, we removed all dolomite-treated plots and all flooded plots from the revised analyses. The revised manuscript now reflects this reduced dataset throughout. The main results remained unchanged. We believe this change makes the inference cleaner and avoids over-interpreting a confounded dolomite treatment.

## Methods citations and transparency.

*Several sections, particularly within the Methods, contain limited citation of supporting literature. The manuscript would benefit from stronger reference support for methodological decisions.*

**Response:** We thank the reviewer for this suggestion. We added references in the Methods where appropriate, especially for agroecological management, agronomic efficiency, and economic evaluation. We also clarified procedural details directly in the text when no single citation was especially appropriate. We hope these revisions improve methodological transparency and reproducibility.

## 1. Add information on frass productivity derived from similar waste streams or from the local region.

**Response:** We added a new paragraph in the Introduction describing the expected quantity of frass generated by BSF systems processing organic waste streams, with specific reference to agro-industrial substrates such as brewery spent grain. This addition is intended to better illustrate the potential abundance of frass as a locally available fertilizer alternative.

## 2. Clarify whether the selected frass application rates were based on established references or prior studies.

**Response:** We clarified the rationale for the selected application rates in the Methods. Briefly, the twelve doses were chosen to sample a broad range of the parameter space while concentrating more observations in the intermediate range, where we expected the most informative part of the dose–response curve. The design was also constrained by available field area and replication requirements.

## 3–4. Provide references to support the technical procedures used for planting and for other procedures in the study.

**Response:** We added references where appropriate and clarified the procedures in the Methods. Some measurements, such as germination, vegetative growth, and reproductive status, are standard agronomic observations and were included primarily to complement the main yield-focused analyses.

## 5. Clarify equation parameters and units, including t ha⁻¹.

**Response:** We clarified the notation and units associated with all equation terms in the revised manuscript, including the definition of t ha⁻¹.

## 6. Add a citation to the NAE equation.

**Response:** We added a citation for the NAE equation (Baligar et al., 2001).

## 7. Clarify the NAE formulation and define all variables and units.

**Response:** We clarified the NAE equation, added the supporting citation, and explicitly defined all variables and units in the text. We also removed the NAE-adjusted yield analysis in response to Reviewer #2, which resolved the reviewer’s additional concern regarding that derived metric.

## 8–9. Add a citation to the MVCR equation and support the formula.

**Response:** We added a citation for the MVCR equation (Ragasa et al., 2025) and retained supporting references for commonly used profitability thresholds (Chamberlin et al., 2021; Sheahan et al., 2013; Theriault et al., 2018).

## 10. Clarify the reported application rate associated with the highest NAE.

**Response:** We agree that this value could be confusing. It was model-derived rather than one of the experimental treatment levels. To avoid ambiguity, we replaced it with 0.4 t ha⁻¹, which corresponds to the lowest applied treatment rate. This change does not alter the interpretation.

## 11. Provide a verifiable source for local crop prices described as approximately double the FAO values.

**Response:** We agree that the local market price statement was insufficiently documented. We therefore removed that unsupported comparison from the revised manuscript and retained the FAO-based analysis, which is fully referenced. We also added discussion clarifying how the MVCR framework can be rescaled if local crop prices differ from FAO values.

## 12. Add supporting literature for the statement on the negative effects of excessive fertilizer use.

**Response:** We added supporting citations for the environmental consequences of excessive fertilizer use, including Carpenter et al. (1998), Guo et al. (2010), and Vitousek et al. (1997).

## 13. Provide a citation for the cattle manure price comparison in Madagascar.

**Response:** We removed the comparison with cattle manure in Madagascar because we could not provide a sufficiently verifiable citation. We retained the comparison with mineral NPK fertilizer and now cite a government-backed source for the current price.

## 14. Adjust Figure 4 contour labels to improve readability.

**Response:** Done. The contour labels in Figure 4 were repositioned to improve clarity and reduce overlap.

## 15. Figure 4C is not mentioned in the manuscript text.

**Response:** We removed the cattle manure results, and Figure 4 now presents only the FAO-based analyses discussed in the main text.

## 16. Add more references from the last five years.

**Response:** We added 27 new references, including 11 published within the last five years. We aimed to balance recent literature with older seminal papers that remain important for framing the study.

# Journal requirements and editor comments

## 1. Please ensure that the manuscript meets PLOS ONE style requirements, including file naming.

**Response:** We reviewed the manuscript files and formatting against the PLOS ONE guidelines and will submit the revision using the required file structure and file names.

## 2. Please provide additional information regarding permits obtained for the work.

**Response:** We revised the Methods to clarify field site access and permitting information, including the approving authority where applicable.

## 3. Please remove references to unpublished work from the reference list and instead cite them in the text as unpublished.

**Response:** We revised the manuscript accordingly and removed unpublished items from the reference list where required by the journal guidelines.

## 4. Please include captions for Supporting Information files at the end of the manuscript and update in-text citations.

**Response:** We added captions for the Supporting Information files at the end of the manuscript and updated the corresponding in-text citations.

## 5. Please evaluate reviewer-suggested citations for relevance.

**Response:** We reviewed the suggested publications and incorporated relevant recent references where appropriate, including recent papers on BSF-related literature.

## Editor comment: add recent studies related to the effect of various feeds on BSF larvae, especially the suggested 2024 and 2025 papers.

**Response:** We reviewed the suggested studies and incorporated relevant recent literature to strengthen the manuscript’s framing and discussion.
