# Supplementary material for: Optimizing Black Soldier Fly frass fertilizer rates for maize and soybean production in Madagascar
Source: PLoS One. 2026 May 4;21(5):e0343378. doi: 10.1371/journal.pone.0343378 (PMC13138682; doi:10.1371/journal.pone.0343378)
Supplement: Attachment — Submitted filename: Reviewer #3 Comments - 2nd.docx [file pone.0343378.s006.docx]

Thank you for the revisions and for carefully addressing the previous comments. I appreciate the improvements made, even though some aspects required removal, and I acknowledge that the manuscript has improved overall. However, several points still require further clarification and refinement to strengthen the manuscript. Please consider the following detailed comments (highlighted in yellow for clarity) to further improve the quality, transparency, and robustness of the study.

1. Add information on frass productivity derived from similar waste streams or from the local region.
Response: We added a new paragraph in the Introduction describing the expected quantity of frass generated by BSF systems processing organic waste streams, with specific reference to agro-industrial substrates such as brewery spent grain. This addition is intended to better illustrate the potential abundance of frass as a locally available fertilizer alternative.

Comment: Thank you for the clarification and the additional paragraph. However, the references provided primarily justify frass yield potential based on BSF bioconversion efficiency. To strengthen the argument regarding real-world availability, I encourage the authors to include supporting data from official and region-specific sources (e.g., national statistics agencies or government reports) that quantify the production of relevant organic waste streams (such as agro-industrial residues) in the study area. This would provide a more robust and context-specific basis for assessing the practical abundance and scalability of frass as a fertilizer alternative.

2. Clarify whether the selected frass application rates were based on established references or prior studies.

Response: We clarified the rationale for the selected application rates in the Methods. Briefly, the twelve doses were chosen to sample a broad range of the parameter space while concentrating more observations in the intermediate range, where we expected the most informative part of the dose–response curve. The design was also constrained by available field area and replication requirements.

Comment: Thank you for clarifying the rationale behind the selection of application rates. I understand the constraints and the logic of your experimental design. However, I would kindly request that you also provide references to previous studies or established fertilization practices for this crop. This will help ensure that the tested rates are relatable to common field applications and farmer practices, thereby strengthening the practical relevance of your findings.

3–4. Provide references to support the technical procedures used for planting and for other procedures in the study.

Response: We added references where appropriate and clarified the procedures in the Methods. Some measurements, such as germination, vegetative growth, and reproductive status, are standard agronomic observations and were included primarily to complement the main yield-focused analyses.

Comment: Thank you for adding references and clarifying the procedures. However, I noticed that the citation was only provided at the beginning of the Methods section (“The experiment followed agroecological management practices [5, 26–29]”). For greater clarity and traceability, it would be more effective to include specific references within each relevant subsection, for example, under *Germination*, *Vegetative growth and reproductive development*, *Harvest and yield determination*, and *Germination and growth*. This will allow readers to easily follow and verify the procedures against prior studies, thereby strengthening the methodological rigor of your manuscript.

5. Clarify equation parameters and units, including t ha⁻¹.

Response: We clarified the notation and units associated with all equation terms in the revised manuscript, including the definition of t ha⁻¹.

Comment: Thank you for the clarification.

6. Add a citation to the NAE equation.

Response: We added a citation for the NAE equation (Baligar et al., 2001).

Comment: Thank you for adding the citation for the NAE equation. The reference is appropriate; however, the phrase “(Equation 1)” should still be explicitly included in the text. The intended format is: *“Nitrogen agronomic efficiency (NAE) was calculated as (Equation 1):”* followed by the citation (e.g., Baligar et al., 2001). Please see, for example, the formatting used in [https://doi.org/10.1371/journal.pone.0332046]. Including both the equation label and the reference will improve clarity and consistency for readers.

7. Clarify the NAE formulation and define all variables and units.

Response: We clarified the NAE equation, added the supporting citation, and explicitly defined all variables and units in the text. We also removed the NAE-adjusted yield analysis in response to Reviewer #2, which resolved the reviewer’s additional concern regarding that derived metric.

Comment: Thank you for the clarification. This revision is reasonable and can be accepted.

8–9. Add a citation to the MVCR equation and support the formula.

Response: We added a citation for the MVCR equation (Ragasa et al., 2025) and retained supporting references for commonly used profitability thresholds (Chamberlin et al., 2021; Sheahan et al., 2013; Theriault et al., 2018).

Comment: Thank you for adding the citation for the MVCR equation and supporting references. However, as indicated in my earlier point (see Comment 6), the phrase “(Equation XX)” should still be explicitly included in the text after *“Economic performance was evaluated using the marginal value-cost ratio (MVCR), calculated as”*. This formatting, together with the citation, will ensure clarity and consistency for readers.

10. Clarify the reported application rate associated with the highest NAE.

Response: We agree that this value could be confusing. It was model-derived rather than one of the experimental treatment levels. To avoid ambiguity, we replaced it with 0.4 t ha⁻¹, which corresponds to the lowest applied treatment rate. This change does not alter the interpretation.

Comment: Thank you for the clarification. This revision is reasonable and can be accepted.

11. Provide a verifiable source for local crop prices described as approximately double the FAO values.
Response: We agree that the local market price statement was insufficiently documented. We therefore removed that unsupported comparison from the revised manuscript and retained the FAO-based analysis, which is fully referenced. We also added discussion clarifying how the MVCR framework can be rescaled if local crop prices differ from FAO values.

Comment: Thank you for the clarification. This revision is reasonable and can be accepted.

12. Add supporting literature for the statement on the negative effects of excessive fertilizer use.
Response: We added supporting citations for the environmental consequences of excessive fertilizer use, including Carpenter et al. (1998), Guo et al. (2010), and Vitousek et al. (1997).

Comment: Thank you for adding the supporting citations. The references provided are relevant, however, it would further strengthen the manuscript if more recent studies could also be included alongside the classic works.

13. Provide a citation for the cattle manure price comparison in Madagascar.

Response: We removed the comparison with cattle manure in Madagascar because we could not provide a sufficiently verifiable citation. We retained the comparison with mineral NPK fertilizer and now cite a government-backed source for the current price.

Comment: Thank you for the clarification. This revision is reasonable and can be accepted.

14. Adjust Figure 4 contour labels to improve readability.

Response: Done. The contour labels in Figure 4 were repositioned to improve clarity and reduce overlap.

Comment: Thank you for revising Figure 4. The repositioning of the contour labels has clearly improved readability, and the graph is now much easier to interpret.

15. Figure 4C is not mentioned in the manuscript text.

Response: We removed the cattle manure results, and Figure 4 now presents only the FAO-based analyses discussed in the main text.

Comment: Thank you for the clarification.

16. Add more references from the last five years.

Response: We added 27 new references, including 11 published within the last five years. We aimed to balance recent literature with older seminal papers that remain important for framing the study.

Comment: Thank you for adding the additional references, including several recent ones. However, the manuscript still appears to rely heavily on citations older than five years. I leave it to the editor’s discretion to determine whether the current balance between recent and older references is acceptable or if further adjustment is required.
