# Supplementary material for: Optimizing Black Soldier Fly frass fertilizer rates for maize and soybean production in Madagascar
Source: PLoS One. 2026 May 4;21(5):e0343378. doi: 10.1371/journal.pone.0343378 (PMC13138682; doi:10.1371/journal.pone.0343378)
Supplement: Attachment — Submitted filename: Response_to_Reviewers_auresp_2.docx [file pone.0343378.s008.docx]

6. Review Comments to the Author

Please use the space provided to explain your answers to the questions above. You may also include additional comments for the author, including concerns about dual publication, research ethics, or publication ethics. (Please upload your review as an attachment if it exceeds 20,000 characters)

Reviewer #1: Paper could be accepted for the publication. So, I can suggest for the publication.

Response: We appreciate the reviewer’s positive evaluation.

Best Regards.

Reviewer #2: The authors have performed a thorough revision. The most critical changes: removing the confounded dolomite and flood-affected plots and eliminating the conceptually weak "NAE-adjusted yield" analysis, demonstrate a strong commitment to scientific rigor and clarity. The manuscript is now more focused, with a cleaner narrative centered on biophysical response and economic profitability.

The authors have not only made the requested changes but have gone further to simplify and strengthen the manuscript. The revised version is significantly improved in terms of methodological transparency, conceptual clarity, and interpretive caution.

I would, however, suggest for the authors to conduct a thorough peer review to check the terminologies and check if there are any typographical errors to improve the manuscript flow and readability. After that, I believe the manuscript is fit for publication. Congratulations.

Response: We thank the reviewer for their valuable comments, which significantly improved the manuscript. We have expanded and updated the citations (see Reviewer #3) and confirmed the consistency and accuracy of the terminology.

Reviewer #3: Thank you for the revisions and for carefully addressing the previous comments. I appreciate the improvements made, even though some aspects required removal, and I acknowledge that the manuscript has improved overall. However, several points still require further clarification and refinement to strengthen the manuscript. Please consider the following detailed comments (highlighted in yellow for clarity) to further improve the quality, transparency, and robustness of the study.

1. Add information on frass productivity derived from similar waste streams or from the local region.
Response: We added a new paragraph in the Introduction describing the expected quantity of frass generated by BSF systems processing organic waste streams, with specific reference to agro-industrial substrates such as brewery spent grain. This addition is intended to better illustrate the potential abundance of frass as a locally available fertilizer alternative.
Comment: Thank you for the clarification and the additional paragraph. However, the references provided primarily justify frass yield potential based on BSF bioconversion efficiency. To strengthen the argument regarding real-world availability, I encourage the authors to include supporting data from official and region-specific sources (e.g., national statistics agencies or government reports) that quantify the production of relevant organic waste streams (such as agro-industrial residues) in the study area. This would provide a more robust and context-specific basis for assessing the practical abundance and scalability of frass as a fertilizer alternative.

Response: We have rewritten the entire paragraph to better explain the potential of BSF production systems in Madagascar. We have included two new data points on the quantities of industrial and domestic waste that could be used as feedstock. As BSF farming is a relatively recent practice in Madagascar, there are currently no published data on the actual number of BSF farms.

The paragraph now reads:

Black Soldier Fly (BSF) production systems can generate substantial quantities of

frass relative to the volume of organic waste processed [15]. Depending on rearing

conditions and feedstock composition, BSF larvae typically convert 15–25% of the initial

waste mass into larval biomass, while 40–60% remains as residual frass and substrate

material [26, 27]. In Madagascar, BSF rearing systems have access to large and

continuous feedstock streams. Agro-industrial by-products constitute a primary source.

For example, STAR Madagascar produces over 90,000 tons of brewery spent grain

annually [28], part of which is currently valorized by EXA Feed [29, 30]. A second

important source is food waste, estimated at around 15% for Madagascar [31]. In urban

areas, these organic waste streams have already been leveraged for industrial-scale BSF

production, as illustrated by initiatives such as BSF Tamatave [32, 33]. In rural areas,

similar organic waste could support smaller-scale, family-operated production systems.

Recent evidence indicates a high level of acceptance of insect-based products among the

population [34]. In addition, the establishment of national standards authorizing BSF

larvae and derived products for human consumption may further incentivize the

expansion of BSF farming [35]. This growth is expected to generate increasing quantities

of frass, with potential applications as an organic fertilizer in agricultural systems.

2. Clarify whether the selected frass application rates were based on established references or prior studies.
Response: We clarified the rationale for the selected application rates in the Methods. Briefly, the twelve doses were chosen to sample a broad range of the parameter space while concentrating more observations in the intermediate range, where we expected the most informative part of the dose–response curve. The design was also constrained by available field area and replication requirements.
Comment: Thank you for clarifying the rationale behind the selection of application rates. I understand the constraints and the logic of your experimental design. However, I would kindly request that you also provide references to previous studies or established fertilization practices for this crop. This will help ensure that the tested rates are relatable to common field applications and farmer practices, thereby strengthening the practical relevance of your findings.

Response: We agree that contextualizing the tested rates against established references strengthens the Methods. We have added a sentence to the "Experimental design and fertilizer application" subsection explicitly linking our rate range to prior studies and local practice. The low end of our design (0.4–1.2 t ha⁻¹) captures rates comparable to documented smallholder practice in Madagascar ([22], Solofondranohatra et al. 2025) and the minimum doses reported in field-scale BSF frass trials for maize ([15], Beesigamukama et al. 2020). The intermediate range spans the nationally recommended dose for maize (3.6 t ha⁻¹; FOFIFA, [32]) and includes rates consistent with prior trials for soybean ([23], Kigeso et al. 2025; [24], Yudistira et al. 2024). The upper rates (20–30 t ha⁻¹) intentionally exceed published recommendations and prior trial ranges to explore the upper limits of the agronomic response curve.

Text changes in the first paragraph of "Experimental design and fertilizer application":

“....The range of tested rates encompass both locally documented smallholder practice (1.2 t

ha-1; [36]) and the nationally recommended dose for maize (3.6 t ha-1; [44]), while

extending to rates consistent with or exceeding those reported in published BSF frass

field trials for maize [21] and soybean [37, 38]. This design ensures that our

dose–response inferences are grounded in agronomically relevant contexts while

providing sufficient range to characterize the full shape of the response curve….”

3–4. Provide references to support the technical procedures used for planting and for other procedures in the study.
Response: We added references where appropriate and clarified the procedures in the Methods. Some measurements, such as germination, vegetative growth, and reproductive status, are standard agronomic observations and were included primarily to complement the main yield-focused analyses.
Comment: Thank you for adding references and clarifying the procedures. However, I noticed that the citation was only provided at the beginning of the Methods section (“The experiment followed agroecological management practices [5, 26–29]”). For greater clarity and traceability, it would be more effective to include specific references within each relevant subsection, for example, under Germination, Vegetative growth and reproductive development, Harvest and yield determination, and Germination and growth. This will allow readers to easily follow and verify the procedures against prior studies, thereby strengthening the methodological rigor of your manuscript.

Response: We have added these references in each of subsection below and revised the text:

Germination Subsection

Add citations to Solofondranohatra et al. ([36]) and Kigeso et al. ([37]), which use the same germination assessment approach (proportion of emerged seedlings at ~15–16 DAS) in BSF frass trials on the same crops.

"Germination was assessed 16 DAS for maize and 15 DAS for soybean, following the

approach used in comparable BSF frass field trials [36, 37]. For each plot, the proportion

of emerged seedlings relative to the number of seeds sown was recorded”.

Vegetative Growth and Reproductive Development Subsection

Add citation to Zhang et al. ([45]) for maize plant height measurement protocols, and Patjaiko et al. ([46]) for soybean canopy assessment.

Maize: "In maize, vegetative growth was measured 39 DAS, following standard agronomic protocols [45]. Total plant height..."

Soybean: "In soybean, vegetative growth was measured 38 DAS. Vegetative growth was quantified as canopy cover [46], estimated by measuring the maximum plant length and the perpendicular width ..."

Harvest and Yield Determination Subsection

Add citations to Beesigamukama et al. ([21]) for the maize harvest protocol (individual plant harvest, ear measurements, air-drying, threshing) and Kigeso et al. ([37]) or Yudistira et al. ([38]) for soybean pod threshing and dry grain weight.

Maize: "Maize was harvested manually 128 DAS, when all ears had fully matured and dried in the field, following the individual-plant harvest protocol [21]. For each plant..."

Soybean: "Soybean was harvested manually between 90 and 92 DAS, once all pods had dried but before they burst open [37, 38]."

Germination and Growth (Data Analyses) Subsection

Add a citation to Solofondranohatra et al. ([36]) as a comparable field study employing the same GLMM approach for crop germination data.

"The effect of fertilizer application rate on germination was analyzed using generalized linear mixed-effects models (GLMMs) with a binomial error distribution, consistent with the analytical approach used in comparable field studies [36]. The application rate was set as a fixed effect..."

5. Clarify equation parameters and units, including t ha⁻¹.
Response: We clarified the notation and units associated with all equation terms in the revised manuscript, including the definition of t ha⁻¹.
Comment: Thank you for the clarification.

Response: We thank the reviewer for their acknowledgment and are glad that the clarification was helpful.

6. Add a citation to the NAE equation.
Response: We added a citation for the NAE equation (Baligar et al., 2001).
Comment: Thank you for adding the citation for the NAE equation. The reference is appropriate; however, the phrase “(Equation 1)” should still be explicitly included in the text. The intended format is: “Nitrogen agronomic efficiency (NAE) was calculated as (Equation 1):” followed by the citation (e.g., Baligar et al., 2001). Please see, for example, the formatting used in [<https://doi.org/10.1371/journal.pone.0332046>]. Including both the equation label and the reference will improve clarity and consistency for readers.

Response: The text has been revised to comply with the suggested formatting:

“Nitrogen agronomic efficiency (NAE, Equation 1) was calculated following the equation stated by Baligar et al. [2001]: …”

7. Clarify the NAE formulation and define all variables and units.
Response: We clarified the NAE equation, added the supporting citation, and explicitly defined all variables and units in the text. We also removed the NAE-adjusted yield analysis in response to Reviewer #2, which resolved the reviewer’s additional concern regarding that derived metric.
Comment: Thank you for the clarification. This revision is reasonable and can be accepted.

Response: We thank the reviewer for their positive assessment and are pleased that the revision is acceptable.

8–9. Add a citation to the MVCR equation and support the formula.
Response: We added a citation for the MVCR equation (Ragasa et al., 2025) and retained supporting references for commonly used profitability thresholds (Chamberlin et al., 2021; Sheahan et al., 2013; Theriault et al., 2018).
Comment: Thank you for adding the citation for the MVCR equation and supporting references. However, as indicated in my earlier point (see Comment 6), the phrase “(Equation XX)” should still be explicitly included in the text after “Economic performance was evaluated using the marginal value-cost ratio (MVCR), calculated as”. This formatting, together with the citation, will ensure clarity and consistency for readers.

Response: The text has been revised to comply with the suggested formatting:

“MVCR (Equation 2) was calculated using the equation stated by Ragasa et al. [2025]:”

10. Clarify the reported application rate associated with the highest NAE.
Response: We agree that this value could be confusing. It was model-derived rather than one of the experimental treatment levels. To avoid ambiguity, we replaced it with 0.4 t ha⁻¹, which corresponds to the lowest applied treatment rate. This change does not alter the interpretation.
Comment: Thank you for the clarification. This revision is reasonable and can be accepted.

Response: Thank you

11. Provide a verifiable source for local crop prices described as approximately double the FAO values.
Response: We agree that the local market price statement was insufficiently documented. We therefore removed that unsupported comparison from the revised manuscript and retained the FAO-based analysis, which is fully referenced. We also added discussion clarifying how the MVCR framework can be rescaled if local crop prices differ from FAO values.
Comment: Thank you for the clarification. This revision is reasonable and can be accepted.

Response: Thank you

12. Add supporting literature for the statement on the negative effects of excessive fertilizer use.
Response: We added supporting citations for the environmental consequences of excessive fertilizer use, including Carpenter et al. (1998), Guo et al. (2010), and Vitousek et al. (1997).
Comment: Thank you for adding the supporting citations. The references provided are relevant, however, it would further strengthen the manuscript if more recent studies could also be included alongside the classic works.

Response: We have incorporated four recent references (published within the last five years) to better document the negative impacts of mineral fertilizers, including their contribution to greenhouse gas emissions. The text now reads:

“In addition, the long-term and often excessive use of mineral fertilizers in high-input systems has been associated with soil degradation, nutrient imbalances, water pollution, and greenhouse gas emissions [7–13].”

13. Provide a citation for the cattle manure price comparison in Madagascar.
Response: We removed the comparison with cattle manure in Madagascar because we could not provide a sufficiently verifiable citation. We retained the comparison with mineral NPK fertilizer and now cite a government-backed source for the current price.
Comment: Thank you for the clarification. This revision is reasonable and can be accepted.

Response: Thank you

14. Adjust Figure 4 contour labels to improve readability.
Response: Done. The contour labels in Figure 4 were repositioned to improve clarity and reduce overlap.
Comment: Thank you for revising Figure 4. The repositioning of the contour labels has clearly improved readability, and the graph is now much easier to interpret.

Response: We thank the reviewer for their positive assessment and are pleased that the revised figure has improved readability.

15. Figure 4C is not mentioned in the manuscript text.
Response: We removed the cattle manure results, and Figure 4 now presents only the FAO-based analyses discussed in the main text.
Comment: Thank you for the clarification.

Response: Thank you

16. Add more references from the last five years.
Response: We added 27 new references, including 11 published within the last five years. We aimed to balance recent literature with older seminal papers that remain important for framing the study.
Comment: Thank you for adding the additional references, including several recent ones. However, the manuscript still appears to rely heavily on citations older than five years. I leave it to the editor’s discretion to determine whether the current balance between recent and older references is acceptable or if further adjustment is required.

Response: We did our best to ensure that all citations added in this revision were published within the last five years. We believe that the updated bibliography reflects the current state of the field; indeed, all BSF-related references are from the last five years. Older citations have been retained primarily for seminal works and established methodological references.
